# Floristic diversity and vegetation of the az Zakhnuniyah Island, Arabian Gulf, Saudi Arabia

**DOI:** 10.1016/j.heliyon.2022.e09996

**Published:** 2022-07-19

**Authors:** Wafa’a A. Al-Taisan

**Affiliations:** Department of Biology, College of Science, Imam Abdulrahman Bin Faisal University, Dammam, 31441, Saudi Arabia

**Keywords:** Biodiversity, Cluster analysis, Conservation, Halophytes, Salt marshes, Ordination

## Abstract

Islands are broadly recognized as hotspots of ecology, biological and geophysical diversity with unique plant species. The present study aimed to address the floristic composition in the Az Zakhnuniyah Island along the Arabian Gulf of Saudi Arabia. A total of 21 quadrats, of 100 m^2^ each were sampled, and both relative density and cover were determined. Classification (Agglomerative Hierarchical Clustering {AHC} and ordination [Detrended Correspondence Analysis (DCA) and Canonical Correspondence Analysis (CCA)] were applied to identify vegetation clusters and their correlation to the underlying soil factors. Fifty plant species belonging to 21 families were recorded. Amaranthaceae (22.9%), Poaceae (12.5%), Asteraceae and Zygophyllaceae (8.33% each) were the largest represented families. Therophytes and chamaephytes were the most represented life-forms, indicating saline-desert vegetation. Most of the surveyed plant species are used for medicinal purposes and grazing. AHC and DCA allowed identifying three vegetation clusters within three distinct habitats: cluster (A): *Halopeplis perfoliata*- *Suaeda vermiculata* in wet salt-marsh habitat, cluster (B): *Limonium axillare*- *Zygophyllum mandaville* in sabkha, and cluster (C): *Heliotropium bacciferum*- *Panicum turgidum* in sand dune habitat within the island. The CCA results imply strong relationships between floristic composition and salinity measures (CaCO_3_, electric conductivity, Mg^+2^, Na^+^, K^+^, Cl^−^ and SO_4_) and soil texture. The current vegetation pattern in Zakhnuniyah Island reflects a salinity gradient with variations in soil texture. Knowledge of the floristic composition and its correlation to the environmental factors within islands should guide future conservation strategy and management efforts.

## 1 Introduction

Distribution patterns and structure of vegetation are the main components in the function of coastal ecosystems that provide different ecosystem services across the globe [1]. Instead, the coastal vegetation is still suffering several threats, for example, grazing, trampling, urbanization, reclamation, pollution, climate change impacts [2]. In recent years, the floristic composition and its environmental factors have become important issues, particularly when conserving and managing plant diversity in coastal arid regions [3, 4, 5]. Plant community distribution and structure in various coastal ecosystems are controlled by their responses to environmental factors, such as topography, salinity, available water, and soil features Therefore, plant diversity differs within dissimilar habitats in the same ecosystem [6].

The Arabian Gulf is a shallow sea characterized by high levels of seawater temperature and salinity [7]. High temperatures and shallow waters are found particularly on the west coast in front of the Gulf’s states. Despite the extreme climatic conditions and harsh environmental conditions, the Arabian Gulf nevertheless enjoys a great diversity of environmental and biological habitats [8, 9]. The saline substratum of the Arabian Gulf land is optimal for the growth of salt-tolerant plants [10]. Saudi Arabia is characterized by harsh desert environments with the absence of rivers or lakes [11]. It is differentiated into various ecosystems, including sandy deserts, mountains, wadis, meadows and saltpans [12].

Saline coasts, particularly, are formed everywhere on the earth, under arid and semi-arid regions [13]. In Saudi Arabia, the salt-affected regions are distinguished into the coastal plain, saline habitats, and littoral salt marshes [14]. The coastal plain is dominated by halophytic communities, which bioaccumulate salts and help in phytoremediation [15]. The highly stressful coastal and inland saline habitats are called sabkhas. These sabkhas are wet and highly saline ecosystems where the soil surface is often covered with thick salt crust [16]. On the other hand, salt marshes provide exceptional habitats for few plant species that cannot survive in other environments.

Most of the key species in the saline habitats are perennial halophytes, which comprise about 2% of the world’s flora [17]. Therefore, they are a vital component of our environmental structure. Among others, sabkhas are unique ecosystems that are extremely saline where especially halophytic plants can survive [17]. These halophytes complete their life cycle under high saline conditions [17]. Halophytes have two main systems and mechanisms to handle the saline environment; avoidance and tolerance mechanisms [18]. Differences in soil features, salinity levels, flood frequency, altitude, or hydro-period influence the distribution of vegetation in saline habitats [19, 20].

Islands are broadly recognized as hotspots of plant diversity with unique ecology and geophysical diversity [21, 22, 23]. Although Zakhnuniyah Island is one of the most diverse islands with unique ecological and heritage features in the Arabian Gulf, there had been no previous studies that addressed its vegetation and environmental factors. In addition, quantitative floristic studies that investigate the plant communities’ structure and their correlations with ecological factors, particularly, in unique ecosystems are necessary to combat biodiversity loss. Such studies are crucial for insular species as well as provide data for endorsing conservation decisions. Hence, this study aimed to (a) document and analyze the floristic composition and its economic importance, and (b) investigate the correlation between soil factors and vegetation composition along Zakhnuniyah Island.

## 2 Materials and methods

### 2.1 Study area

**Figure 1:**
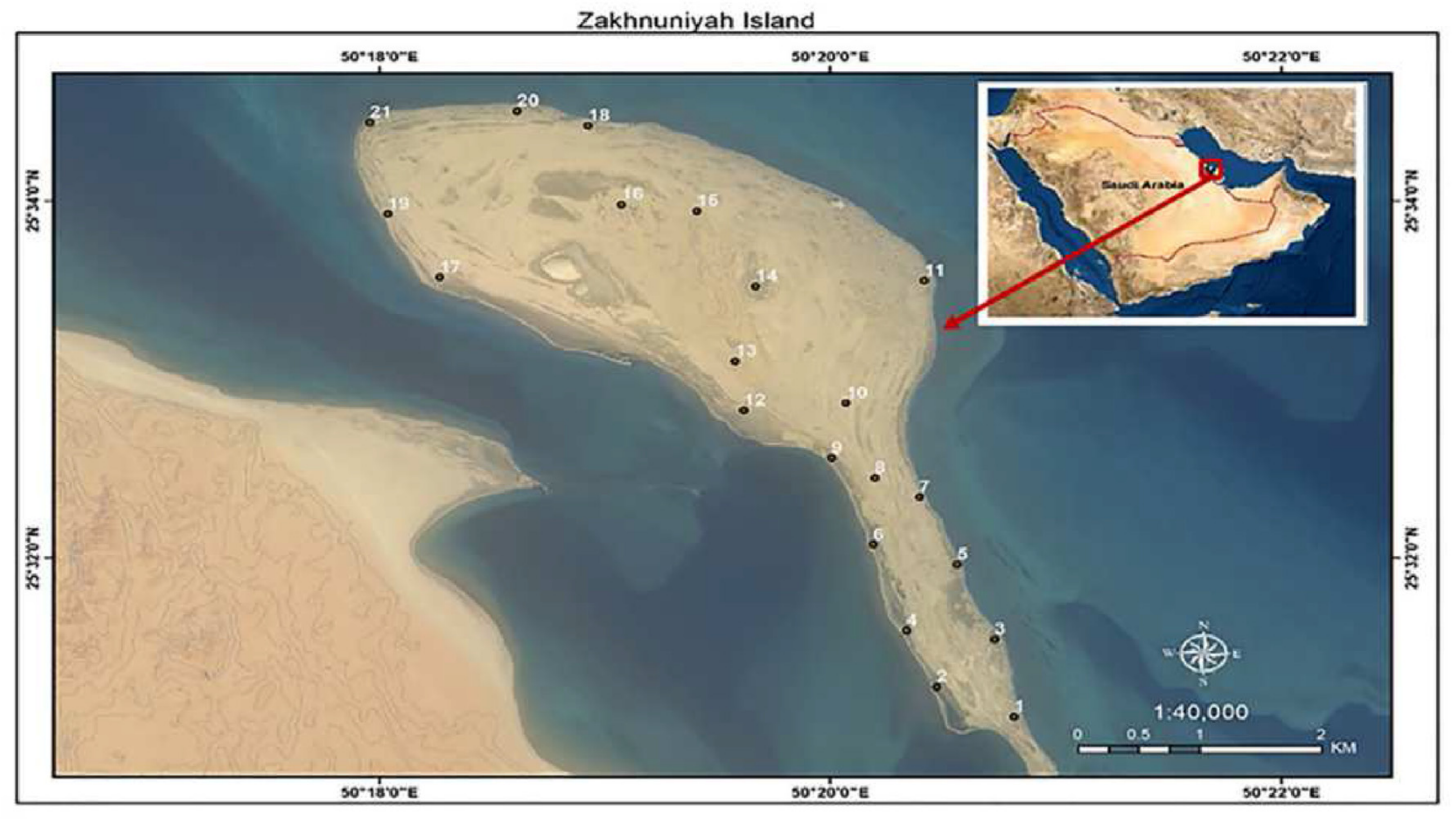
Location map and distribution of quadrats (1-21) within the Zakhnuniyah Island, Saudi Arabia.

The current study was conducted on Az Zakhnuniyah (Jazirat az Zakhnuniyah) Island. Zakhnuniyah is an island located on the western coast of the Arabian Gulf (N 25°54'72.94", E 50° 32'53.31") [24] (Figure 1). The total area of the island is 13.35 km^2^ (1335 ha). The island is characterized by sand dunes, salt marshes, sabkhas, and clay soils. In addition, it is featured by biological diversity including halophytic/xerophytic plants, seagrass, birds, fishes, algae, jellyfish and corals reefs. The main activities on the island are fishing and tourism which led to vegetation degradation. The study area has a subtropical arid climate, dust storms and sandstorms. Temperature exceeds 46 °C during the summer [25], while winter is a short period, and the mean minimum temperature reaches 15.6 °C, with the coldest mean temperatures in January and warmest in July and August. The average annual temperature is 20.1 °C. The rainy season is limited from November to May. The average annual rainfall is ≈74.0 mm/year and mostly falls during the winter months .

### 2.2 Vegetation sampling

The vegetation sampling was carried out between November 2020 and April 2021 with several field trips to Zakhnuniyah Island. For vegetation sampling, 21 quadrats (each of 10 m × 10 m) under random sampling were selected. The vegetation on the island is discontinuous with patchy distribution, therefore, the distribution and number of sampling quadrats were based on the floristic and ecological variability. All plant species were recorded and identified according to [26, 27]. The plant cover percentage was visually estimated following the cover classes of Braun-Blanquet [28], while density was determined for each species according to [29], and finally, the importance value (out of 200) for each species was calculated by the summation of both relative cover and density. The species’ life-forms were determined according to [30]. According to [31], the chorotypes of all plant species were created to assign them to the corresponding world geographical groups. The economic importance of the surveyed species in the study island was documented according to field observations and literature.

### 2.3 Soil sampling and analysis

For each quadrate, composite soil samples (21 quadrates × 3 samples) were randomly collected from a depth of 0–30 cm. The hydrometer method was used to find out the texture of the soil [32]. The calcimeter method was used to determine the calcium carbonate (CaCO_3_) content [33]. A soil water extract (1:5 w/v) was prepared for the determination of pH and electrical conductivity (EC). The concentrations of Cl^−^ and SO_4_ were estimated by the titration method, while the determination of Ca^2+^, Mg^2+^, Na^+^, K^+^, Cu, Zn, Ni and Pb was carried out by atomic absorption spectrophotometer (Shimadzu AA-6200 model, Shimadzu Co., Japan).

### 2.4 Data analysis

The data of the importance values of the plant species within 21 quadrats were subjected to multivariate analysis; using Agglomerative Hierarchical Clustering (AHC) for classification, and Detrended Correspondence Analysis (DCA) for ordination. One-way ANOVA was used to examine the significant difference among soil variables in the identified plant clusters and mean values were separated using the Kruskal-Wallis’s test at p < 0.05. In order to detect the relationship between the dominant and important plant species of the identified plant clusters on one hand and soil variables on the other hand, Canonical Correspondence Analysis (CCA) was applied [34]. Classification, ordination and statistical analyses were performed using XLSTAT (v. 2018, Addinsoft, NY, USA) and MVSP (Version. 3, Kovach Computing Services, Wales, UK).

## 3 Results

### 3.1 Floristic composition in Zakhnuniyah Island

In Zakhnuniyah Island, a total of 50 plant species belonging to 46 genera and 22 families were recorded (Table 1). The surveyed plant species are distinguished into 22 perennials and 28 annuals. The most highly represented families were Amaranthaceae (11 species, 22% of the total recorded species), followed by Poaceae (seven species, 14%), and Asteraceae and Zygophyllaceae (four species each, 8.33% each).

**Table 1:** Floristic composition of Zakhnuniyah Island.

Species	Family	Life-form	Growth-form	Chorotype	Use
**Perennials**					
*Aeluropus lagopoides* (L.) Thwaites	Poaceae	G	Grass	IT + SA	Grazing
*Anabasis setifera* Maq.	Amaranthaceae	Ch	Shrub	SA	Medicinal & grazing
*Arthrocnemum macrostachyum* (Moric.) Piirainen & G. Kadereit	Amaranthaceae	Ch	Shrub	ME + IT	Grazing
*Astragalus sieberi* DC.	Fabaceae	Ch	Shrub	SA	Medicinal
*Caroxylon imbricatum* Forssk.	Amaranthaceae	Ch	Shrub	SA + SU	Medicinal& grazing
*Cenchrus divisus* (J. Gmel.) Verloove	Poaceae	H	Grass	SA + SU	Grazing
*Cressa cretica* L.	Convolvulaceae	H	Shrub	ME + IT	Medicinal& grazing
*Cyperus conglomeratus* Rottb.	Cyperaceae	G	Herb	SA	Medicinal& grazing
*Fagonia indica* Burm. f.	Zygophyllaceae	Ch	Herb	SA	Medicinal
*Halocnemum strobilaceum* (Pall.) M. Bieb.	Amaranthaceae	Ch	Shrub	ME + IT	Medicinal
*Halopeplis perfoliata* (Forssk.) Bunge ex Asch.	Amaranthaceae	Ch	Shrub	ME + IT	Other use
*Haloxylon salicornicum* (Moq.) Bunge ex Boiss.	Amaranthaceae	Ch	Shrub	IT	Medicinal& grazing
*Heliotropium bacciferum* Forssk.	Boraginaceae	Ch	Herb	SA	Medicinal
*Juncus rigidus* Desf.	Juncaceae	G	Grass	IT + SA	Medicinal& grazing
*Lasiurus scindicus* Henr.	Poaceae	G	Grass	SU	Grazing
*Leptadenia pyrotechnica* Forssk.) Decne.	Apocynaceae	Ph	Shrub	SA + SU	Medicinal
*Limonium axillare* (Forssk.) Kuntze	Plumbaginaceae	H	Shrub	SA	Medicinal
*Panicum turgidum* Forssk.	Poaceae	H	Grass	SA + SU	Grazing
*Salsola drummondii* Ulbr.	Amaranthaceae	Ch	Shrub	SA	Grazing
*Suaeda vermiculata* Forssk. ex J.F.Gmel*.*	Amaranthaceae	Ch	Shrub	SA	Medicinal
*Zygophyllum mandavillei* Hadidi	Zygophyllaceae	Ch	Shrub	SA	Medicinal
*Zygophyllum qatarense* Hadidi	Zygophyllaceae	Ch	Shrub	SA	Medicinal
**Annuals**					
*Aizoanthemopsis hispanica* (L.) Klak	Aizoaceae	Th	Herb	ME + SA	Grazing
*Amaranthus viridis* L.	Amaranthaceae	Th	Herb	COSM	Medicinal
*Anastatica hierochuntica* L.	Brassicaceae	Th	Herb	SA	Medicinal
*Aristida mutabilis* Trin.& Rupr.	Poaceae	Th	Grass	TR + SA	Medicinal& grazing
*Cakile arabica* Velen.	Brassicaceae	Th	Herb	ME + IT	Grazing
*Convolvulus rhyniospermus* Choisy	Convolvulaceae	Th	Herb	TR + SA	Other use
*Dipterygium glaucum* Decne	Cleomaceae	Th	Herb	SA + SU	Medicinal& grazing
*Dysphania ambrosioides* (L.) Mosyakin & Clemants	Amaranthaceae	Th	Herb	COSM	Medicinal
*Erodium cicutarium* (L.) L'Hér.	Geraniaceae	Th	Herb	ME + IT	Medicinal
*Euphorbia peplus* L.	Euphorbiaceae	Th	Herb	COSM	Medicinal
*Hordeum murinum* L.	Poaceae	Th	Grass	ME + IT	Grazing
*Launaea capitata* (Spreng.) Dandy	Asteraceae	Th	Herb	ME + SA	Medicinal& grazing
*Lotus halophilus* Boiss*.*	Fabaceae	Th	Herb	ME + SA	Grazing
*Malva parviflora* L.	Malvaceae	Th	Herb	ME + IT	Medicinal &food
*Melilotus indicus* (L.) All	Fabaceae	Th	Herb	PAL	Medicinal
*Moltkiopsis ciliata* (Forssk.) I. M. Johnston	Boraginaceae	Th	Herb	SA	Medicinal
*Neurada procumbens* L.	Neuradaceae	Th	Herb	COSM	Medicinal& grazing
*Paronychia arabica* (L.) DC.	Caryophyllaceae	Th	Herb	SA	Grazing
*Plantago boissieri* Hausskn. & Bornm	Plantaginaceae	Th	Herb	COSM	Grazing
*Poa annua* L.	Poaceae	Th	Grass	COSM	Grazing
*Reseda decursiva* Forssk.	Resedaceae	Th	Herb	SA	Other use
*Rumex vesicarius* L.	Polygonaceae	Th	Herb	SA	Medicinal
*Senecio glaucus* L*.*	Asteraceae	Th	Herb	SA	Grazing
*Silene linearis* Decne	Caryophyllaceae	Th	Herb	SA	Other use
*Sonchus oleraceus* L.	Asteraceae	Th	Herb	COSM	Medicinal
*Suaeda aegyptiaca* (Hasselq) Zoh*.*	Amaranthaceae	Th	Herb	SA	Medicinal
*Tribulus terrestris* L.	Zygophyllaceae	Th	Herb	ME + SU	Medicinal& grazing
*Trigonella anguina* Del.	Fabaceae	Th	Herb	SA	Medicinal

Th: therophyte; Ch: chamaephyte; H: hemicryptophytes; Ph: phanerophytes; G: geophytes; COSM: cosmopolitan; ME: Mediterranean; IT: Irano-Turanian; PAL: Palaeotropical; SA = Saharo-Arabian; SU: Sudano; TR: Tropical.

In regard to the life-forms, the greatest percentage of therophytes (28 species, 56%) was followed by chamaephytes (13 species, 26%), hemicryptophytes (five species, 10%), geophytes (3 species, 6%) and phanerophytes (one species, 2%) (Table 1). The growth habits of the surveyed species were distinguished into 28 herbs (56%), 14 shrubs (28%), and eight grass (16%) (Table 1).

In terms of chorotypes, the floristic structure was mostly made up of Saharo-Arabian species (54%) and Mediterranean species (26%). The Saharo-Arabian element comprised pure Saharo-Arabian (mono-regional with 38%) and bi-regional species with 16%. In addition, the floristic spectrum comprised seven cosmopolitan species.

The economic importance of the recorded species within Zakhnuniyah Island showed that 30% of the total species are used for grazing (e.g. *Aeluropus lagopoides*, *Panicum turgidum*, *Cenchrus divisus* and *Lotus halophilus*), while 60% for medicinal purposes (*Leptadenia pyrotecnica*, *Suaeda vermiculata*, *Chenopodium ambrosioides* and *Anastatica hierochuntica*) and 10% (e.g. *Convolvulus rhyniospermus* and *Reseda decursiva*) for other uses (e.g. dyes, windbreak, sand accumulation, fuel, etc.) (Table 1).

### 3.2 Vegetation clusters

**Figure 2:**
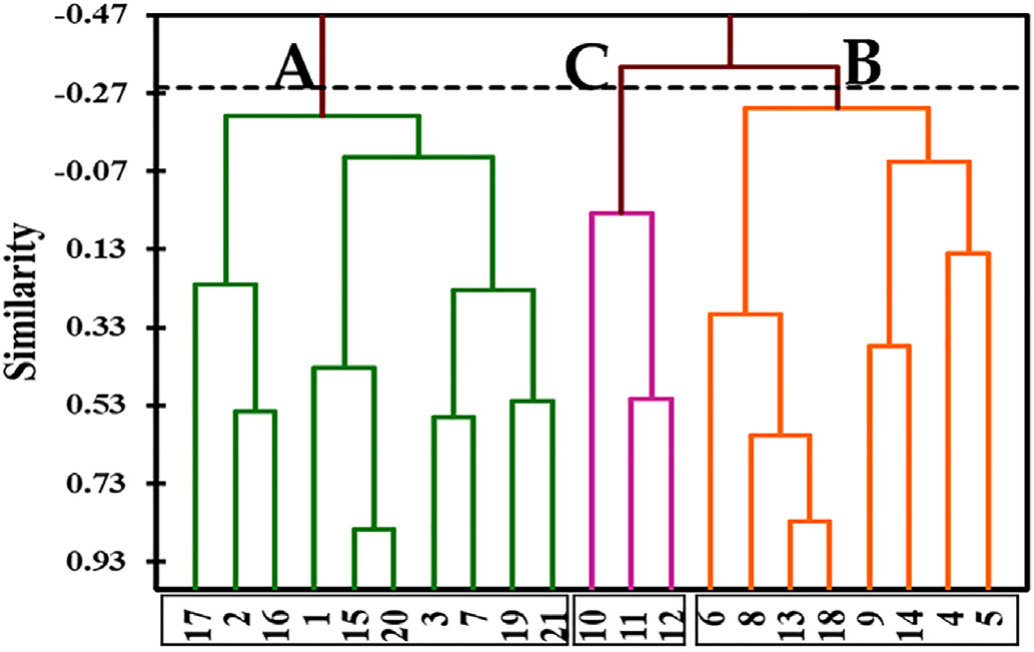
Agglomerative Hierarchical Clustering (AHC) using Pearson-similarity coefficient among sampled quadrats (1-21). Letters (A, B and C) are coded for the vegetation clusters.

The Agglomerative Hierarchical Clustering (AHC) using the Pearson-similarity coefficient among sampled quadrats (1-21) allowed to detect of three vegetation clusters (A, B and C) (Figure 2 & Table 2). These clusters are named according to the two most dominant species with the highest importance values as follows: cluster (A): *Halopeplis perfoliata*- *Suaeda vermiculata*; cluster (B): *Limonium axillare*- *Zygophyllum mandaville*, and cluster (C): *Heliotropium bacciferum*- *Panicum turgidum*.

**Table 2:** Floristic features of the three identified vegetation clusters (A, B and C). The numbers in brackets represent the importance value (out of 200) of each species.

Feature	Vegetation cluster		
	A	B	C
No. of quadrats	10	8	3
Total number of species	31	38	25
Dominant and co-dominant species	*Halopeplis perfoli*ata (41.53)- *Suaeda vermiculata* (31.06)	*Limonium axillare* (45.77)- *Zygophyllum mandaville* (27.37)	*Heliotropium bacciferum* (43.43)- *Panicum turgidum* (22.47)
Important associated species	*Haloxylon salicornicum* (23.85) and *Salsola drummondii* (20.14), *Arthrocnemum macrostachyum* (16.83), *Cressa cretica* (14.11)	*Anabasis lachnantha* (26.36) and *Zygophyllum qatarense* (15.74).	*Cyperus conglomerate* (22.73) and *Pennisetum divisum* (20.52).
Habitat-type	Salt marshes	Sabkhas	Sand dunes

Vegetation cluster (A) comprised 31 species (14 perennials and 17 annuals) distributed within 10 quadrats and occupies salt-marshes habitat within Zakhnuniyah Island (Table 2). This cluster was dominated by *Halopeplis perfoliata* (41.53) and *Suaeda vermiculata* (31.06). The most important associated species in this cluster were *Haloxylon salicornicum* (23.85) and *Salsola drummondii* (20.14). On the other hand, cluster (B) included eight quadrats and 38 species (19 each perennials and annuals) distributed in the sabkhas habitat-type in Zakhnuniyah Island. *Limonium axillare* (45.77) and *Zygophyllum mandaville* (27.37) were the dominant species in cluster (B), while *Anabasis lachnantha* (26.36) and *Zygophyllum qatarense* (15.74) were the important associated species in this cluster. Finally, cluster (C) comprised 25 species (13 perennials and 12 annuals) distributed in three quadrats within the sand-dunes habitat. Moreover, *Heliotropium bacciferum* (43.43) and *Panicum turgidum* (22.47) were the dominant species in this cluster while *Cyperus conglomerate* and *Pennisetum divisum* were the most important associated species (Table 2).

**Figure 3:**
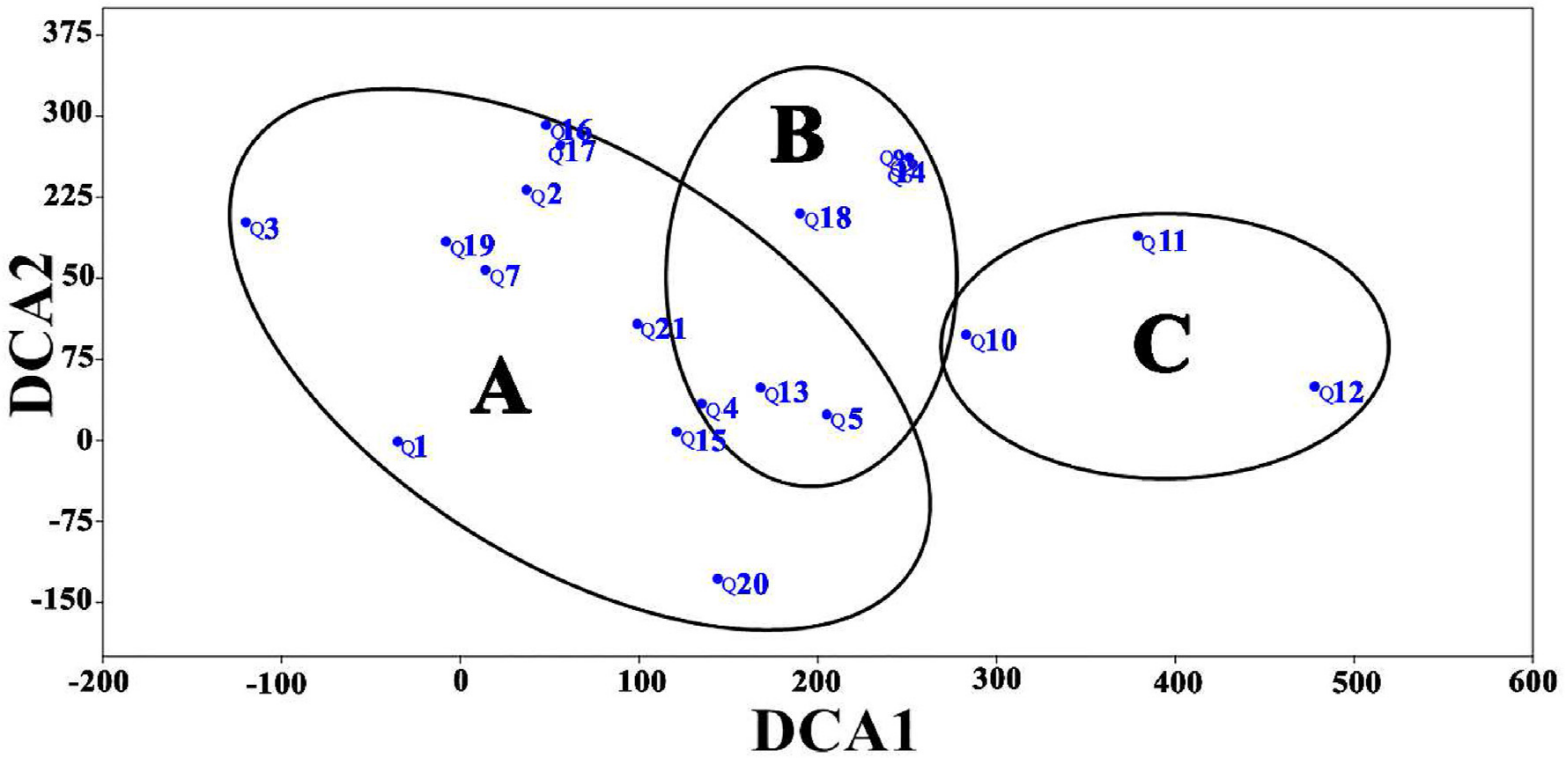
Detrended Correspondence Analysis (DCA) of the three vegetation clusters (A, B and C). Numbers represent the quadrat numbers (Q1-Q21).

On the other hand, the application of Detrended Correspondence Analysis (DCA) ordination on the vegetation data of 21 quadrats confirms the segregation of three vegetation clusters (Figure 3). However, there is an overlapping between clusters (A) and (B), while cluster (C) was separated at the right side of the DCA diagram with a close correlation with cluster (B).

### 3.3 Vegetation clusters-soil correlation

The soil factors of the three identified vegetation clusters are below summarized (Table 3). Except for silt fraction, electric conductivity, Mg, Na, K, Cl, and SO_4_, the other measured soil factors showed no significant differences among the three clusters at p > 0.05. Cluster (A): *Halopeplis perfoliata*- *Suaeda vermiculata* attained the highest values of salinity (CaCO_3_, electric conductivity, Ca, Mg, Na, K, Cl, SO_4_) and Fe, while cluster (B): *Limonium axillare*- *Zygophyllum mandaville* had the maximum values of sand and silt fractions and Mn. The soil of cluster C: *Heliotropium bacciferum*- *Panicum turgidum* had the highest contents of clay fraction, pH, Cu, Zn, Ni and Pb. The soil of cluster (A) had the lowest values of sand, silt, Cu, Zn, Mn and Ni, while the soil of cluster (B) attained the lowest values of clay, pH and Pb. Moreover, the species of cluster (C) inhabit the soils with the lowest contents of salinity and Fe.

**Table 3:** Physical and chemical properties of soil in the three vegetation clusters (A, B and C).

Soil parameter	Vegetation cluster			*p-value*
	A	B	C	
Sand (%)	87.40 ± 8.94^a^	97.25 ± 1.92^a^	91.33 ± 3.17^a^	0.066
Silt (%)	0.40 ± 0.10^a^	0.50 ± 0.30^a^	3.33 ± 0.67^b^	0.008∗
Clay (%)	3.20 ± 1.08^a^	2.25 ± 1.44^a^	5.33 ± 3.33^a^	0.246
CaCO_3_ (%)	15.99 ± 3.69^a^	7.34 ± 1.06^a^	3.37 ± 0.31^a^	0.054
pH	6.69 ± 0.14^a^	6.56 ± 0.21^a^	6.89 ± 0.18^a^	0.213
EC (dS cm^−1^)	88.68 ± 20.05^b^	46.76 ± 11.44^ab^	3.59 ± 0.73^a^	0.045∗
Ca^++^ (meq/L)	1195.22 ± 185.67^a^	934.94 ± 202.76^a^	154.66 ± 19.40^a^	0.054
Mg^++^ (meq/L)	1727.16 ± 489.54^b^	766.22 ± 188.96^ab^	51.27 ± 14.04^a^	0.026∗
Na^+^ (meq/L)	17659.40 ± 4750.40^a^	7856.62 ± 1917.96^a^	275.38 ± 79.41^a^	0.040∗
K^+^ (meq/L)	845.85 ± 215.68^b^	342.69 ± 79.12^ab^	22.23 ± 4.29^a^	0.040∗
Cl^−^ (meq/L)	38657.20 ± 10005.36^b^	16295.30 ± 4228.38^ab^	311.13 ± 89.29^a^	0.031∗
SO_4_^--^ (meq/L)	4546.43 ± 656.80^a^	2954.65 ± 574.21^a^	464.28 ± 118.40^a^	0.040∗
Fe (meq/L)	0.16 ± 0.03^a^	0.06 ± 0.01^a^	0.05 ± 0.01^a^	0.062
Cu (meq/L)	6.28 ± 1.45^a^	7.01 ± 1.19^a^	9.50 ± 2.70^a^	0.613
Zn (meq/L)	13.11 ± 1.24^a^	19.63 ± 3.17^a^	20.82 ± 7.91^a^	0.161
Mn (meq/L)	226.83 ± 29.94^a^	412.04 ± 93.62^a^	237.99 ± 68.92^a^	0.139
Ni (meq/L)	30.06 ± 4.45^a^	35.49 ± 5.29^a^	41.63 ± 16.73^a^	0.530
Pb (meq/L)	0.99 ± 0.28^a^	0.40 ± 0.22^a^	1.19 ± 0.59^a^	0.363

Values are mean ± standard errors. EC: electrical conductivity. Superscript letters within each row showed significant variation at p < 0.05 (Kruskal-Wallis’s test) at df = 2. ∗ significant at p < 0.05.

**Figure 4:**
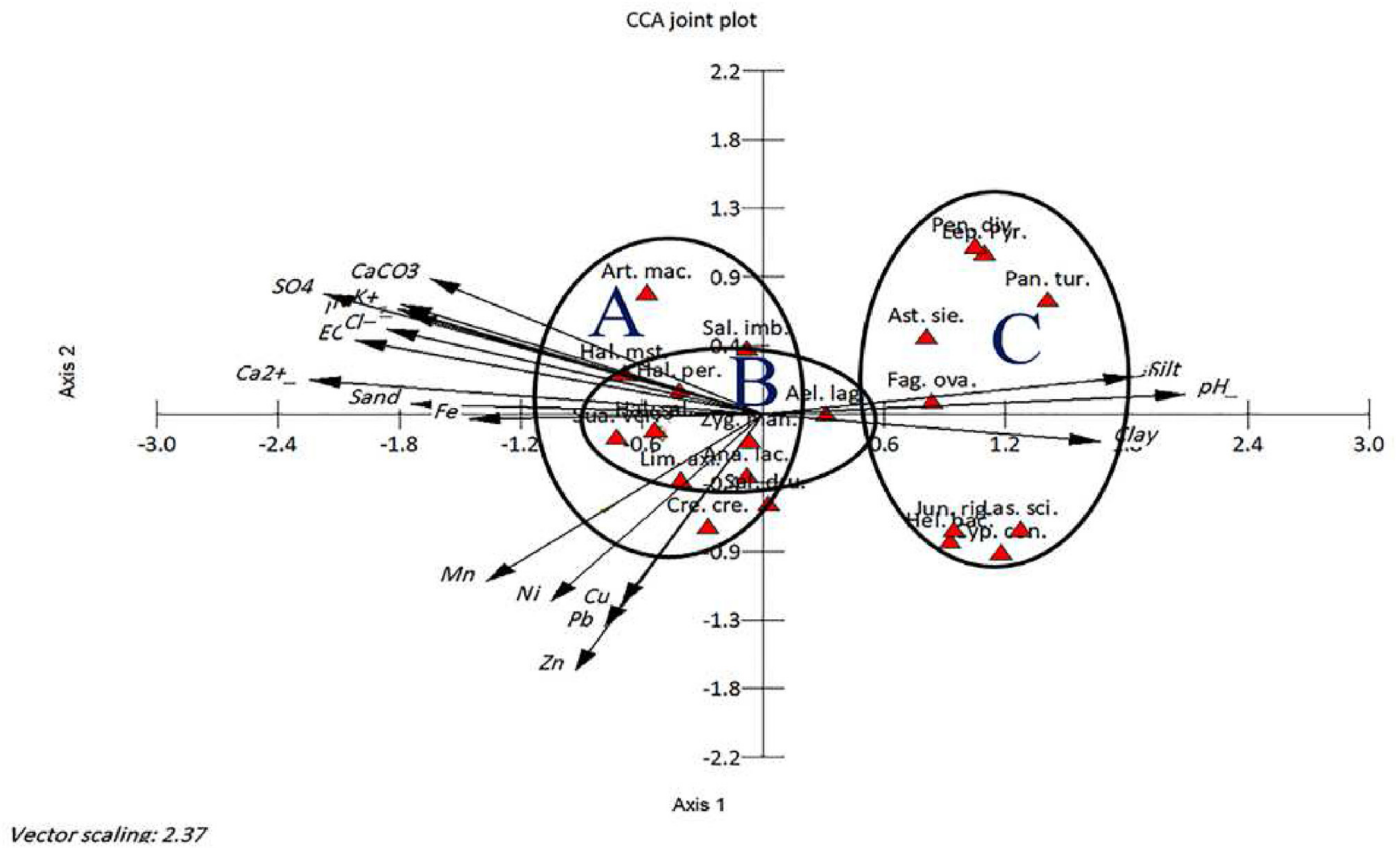
Canonical Correspondence Analysis (CCA) shows the correlation between the soil factors (arrows) and dominant, codominant and important associated species (red triangles) representing the three vegetation clusters (A, B and C). EC: electric conductivity. The species are *Hal per*: *Halopeplis perfoliata*, *Su aver*: *Suaeda vermiculata*, *Hal sal*: *Haloxylon salicornicum*, *Sal dru*: *Salsola drummondii*, *Lim axi*: *Limonium axillare*, *Zyg man*: *Zygophyllum mandaville*, *Ana lac*: *Anabasis lachnantha*, *Zyg qat*: *Zygophyllum qatarense*, *Hel bac*: *Heliotropium bacciferum*, *Pan tur*: *Panicum turgidum*, *Cyp con*: *Cyperus conglomeratus*, *Pen div*: *Pennisetum divisum*, *Ast sie*: *Astragalus sieberi*, *Art mac*: *Arthrocnemum macrostachyum*, *Ael lag*: *Aeluropus lagopoides*, *Cre cri*: *Cressa cretica*, *Jun rig*: *Juncus rigidus*, Lep pyr: *Leptadenia pyrotechnica*.

The application of Canonical Correspondence Analysis (CCA) displayed the correlation between the dominant, co-dominant and important associated species in each identified vegetation cluster and measured soil factors (Figure 4). The CCA biplot showed that cluster C (*Heliotropium bacciferum*- *Panicum turgidum*) was segregated on the right side where it is correlated with pH, silt, and clay. In contrast, cluster A (*Halopeplis perfoliata*- *Suaeda vermiculata*) was separated at the upper left side near the central part of the CCA biplot and showed a close correlation with salinity factors (SO_4_, K, Na, Cl, CaCO_3_, Ca), sand fraction and Fe. On the other hand, cluster B (*Limonium axillare*- *Zygophyllum mandaville*) was segregated at the lower left side near the central part of the CCA biplot and exhibited close correlations with Zn, Mn, Ni, Pb and Cu.

*Halopeplis perfoliata*, the dominant species in the cluster (A) showed positive significant correlations with CaCO_3_, EC, Mg, Na, K, Cl and SO_4_. On the other hand, the first dominant species in cluster B (*Limonium axillare*) displayed a significant negative correlation with Pb. *Heliotropium bacciferum*, the first dominant species in the cluster (C) exhibited positive correlations with Cu, Zn, Ni and Pb and negative correlations with Ca and SO_4_ while *Panicum turgidum* (the codominant species in the same cluster) revealed a positive correlation with silt fraction, and negative correlations with Ca and SO_4_.

## 4 Discussion

As expected, due to the prevailing arid conditions (high temperature, low water availability, wind exposure, and salinity), the Zakhnuniyah Island in the Arabian Gulf, Saudi Arabia only hosts 50 species (21 perennials and 29 annuals). In such conditions, only halophytic or salt-tolerant plants which represent about 1% of the world flora can survive and persist [35]. This could justify the low number of plant species.

In the current study area, the greatest contribution of annuals over perennials may be attributed to their high reproductive and ecological features. In addition, the rainy season provides a better chance for the significant appearance of annuals, which provide a typical physiognomy of their vegetation [36]. This finding agreed with the previous studies of [37] and [38] who confirmed that the key components of vegetation in Saudi Arabia are annual plants.

The life-form offers knowledge that can aid to evaluate the influences of environmental factors on vegetation composition and distribution [3]. In the current study, life-form spectra indicated the preponderance of both therophytes and chamaephytes. This finding can be attributed to high temperature, soil salinity, drought, or topographic variations [39]. The variation in life-form frequency depends on habitat, salinity level, waterlogging and sand deposits. Therophytes are adapted to dry climate, low rainfall and spend an unfavourable season in the seed form [11, 40, 41]. The prevalence of chamaephytes which reflect perennial halophytes can withstand high salt content in both saline habitats and coastal sandy habitats [42]. These results are somewhat similar to floristic spectra in the desert and saline habitats in Saudi Arabia and other arid countries [3, 39, 43, 44, 45, 46, 47, 48].

The predominance of Amaranthaceae and Poaceae in the present study is in harmony with [17] who stated that, Amaranthaceae in eudicots and Poaceae in monocots had the highest number of halophytic genera.

The phytogeographical analysis indicated a distinct dominance of the Saharo-Arabian elements. This finding is supplemented with the location of the study island within the south coastal lowland and subtropical climate [49]. In addition, the Saharo-Arabian plants are considered good indicators for desert habitats. These findings are consistent with other studies on desert and saline habitats in Saudi Arabia [11, 44, 45, 50, 51]. On the other hand, most of the surveyed plant species within Zakhnuniyah Island are used for medicinal purposes and grazing. This finding has also been previously documented for the same species in [52] and [53].

The vegetation of Zakhnuniyah Island was categorized into three vegetation clusters within three distinct habitats (wet salt marshes, sabkhas and sand dunes). Sabkhas habitat is widely distributed along the study island and showed the highest number of plant species followed by salt marshes and sand dunes habitats. The variations in species richness among identified habitats may be attributed to soil variables (e.g. moisture, texture, salinity, organic matter) and microtopography [48, 54]. The dominance of halophytes supports the hypothesis that, habitat types and floristic richness along the island are salinity-dependent. The identified vegetation clusters are comparable to those identified in the Red Sea coastal zones of Saudi Arabia [3, 50, 55]. Along the coastal regions, it is common to find great variations in species richness (number of annuals and perennials) among distinct habitats. This may be justified by the level of salinity, soil moisture and structure, topography and human impacts [16, 17, 56, 57].

Among measured soil factors silt fraction, electric conductivity, Mg, Na, K, Cl, and SO_4_ revealed significant differences among three vegetation clusters and habitats. These differences could be attributed to the effect of salt-spray, soil formations, water-availability, floristic structure, microbes, plant-animal remains and micro-topography [41, 58]. The CCA results displayed strong relationships between floristic composition and salinity and soil texture. Moreover, this finding confirms that, the floristic composition within the study island is salinity-dependent. The growth and abundance of halophytes (e.g. *Halopeplis perfoliata*, *Haloxylon salicornicum*, and *Suaeda vermiculata*) indicate wet saline soils [49]. These species shape the vegetation zonation within the salt marshes and coastal habitats. Along with saline environments, soil salinity is the main ecological feature that affects vegetation composition [3, 59, 60, 61, 62]. In addition, several studies highlighted the importance of soil texture, fertility and pH in controlling vegetation composition and patterns [16, 63, 64].

## 5 Conclusion

The floristic composition in Az Zakhnuniyah Island, Saudi Arabia displayed the presence of 50 plant species belonging to three vegetation clusters and distributed in three distinct habitats (wet salt marshes, sabkhas and sand dunes). The current study revealed variations in species richness and soil features among three habitats. Sabkhas habitat showed high plant diversity compared to the salt marshes and sand dunes habitats. Moreover, the current vegetation patterns reflect a salinity gradient associated with changes in soil texture and nutrients. The current study provides the first quantitative floristic study for Az Zakhnuniyah Island in relation to soil factors, and it highlighted the region's importance in terms of plant diversity. The findings in the current study should support future conservation strategies and guide management efforts.

## Declarations

### Author contribution statement

Wafa’a A. Al-Taisan: Conceived and designed the experiments; Performed the experiments; Analyzed and interpreted the data; Contributed reagents, materials, analysis tools or data; Wrote the paper.

### Funding statement

This research did not receive any specific grant from funding agencies in the public, commercial, or not-for-profit sectors.

### Data availability statement

Data included in article/supp. material/referenced in article.

### Declaration of interest’s statement

The authors declare no conflict of interest.

### Additional information

No additional information is available for this paper.
